# Reflecting on the Diverse Legal Responses to Elder Abuse in the United States

**DOI:** 10.1093/geroni/igaf122.1482

**Published:** 2025-12-31

**Authors:** Erica Costello

**Affiliations:** American Bar Association, Washington, District of Columbia, United States

## Abstract

Elder abuse is a complex and pervasive problem affecting countless older adults throughout the United States. It is estimated that at least one in ten community dwelling older adults have experienced some form of abuse in the past year, including physical abuse, emotional abuse, financial abuse, and neglect by family members or caregivers. This presentation will highlight the diverse legal responses that have been adopted over the past fifty years to reduce and prevent elder abuse, including the creation of elder abuse fatality review teams and multi-disciplinary teams, and the adoption of criminal and civil responses to abuse. Restorative justice practices will also be discussed as a unique viable alternative to criminal or civil responses when older adults are abused by family members or caregivers. Rooted in Native American culture, the concept of restorative justice provides older adults with the opportunity to address elder abuse and resolve challenging disputes within families.

